# Reproductive outcomes after bariatric surgery in women

**DOI:** 10.1007/s00508-021-01986-w

**Published:** 2021-12-08

**Authors:** Dragan D. Micic, Hermann Toplak, Dusan D. Micic, Snezana P. Polovina

**Affiliations:** 1 Department of Medical Sciences, Serbian Academy of Sciences and Arts, Belgrade, Serbia; 2 Universitaetsklinik fur Innere Medizin, Klinische Abteilung für Endokrinologie und Diabetologie, Medizinische Universitat Graz, Auenbruggerplatz 15, 8036 Graz, Austria; 3 Faculty of Medicine, University of Belgrade, Belgrade, Serbia; 4 Clinic for Emergency Surgery, Emergency Center, Clinical Center of Serbia, Belgrade, Serbia; 5 Clinic of Endocrinology, Diabetes and Diseases of Metabolism, Clinical Center of Serbia, Belgrade, Serbia; 6 Faculty of Pharmacy, University Business Academy Novi Sad, Novi Sad, Serbia

**Keywords:** Obesity, Female fertility, Polycystic ovary syndrome (PCOS), Pregnancy, Female sexual function

## Abstract

The presence of obesity may significantly influence female fertility through various mechanisms. Impairment of the hypothalamic-pituitary-ovarian axis in obese women may induce anovulation and infertility. Obesity may have an effect on women’s spontaneous and assisted conception rates, increased miscarriage rates, premature labor, stillbirth and perinatal risks, and menstrual irregularity. It has been suggested that weight loss improves reproductive outcomes due to fertility amelioration and an improvement in menstrual irregularity and ovulation. It is still not known which weight reduction procedures (changes in lifestyle, pharmacological management or bariatric intervention) result in optimal outcome on infertility. Currently, bariatric surgery is defined as the best available method for the management of obesity and its associated diseases.

We have analyzed literature facts about effects of bariatric surgery on the function of the hypothalamic-pituitary-ovarian axis, polycystic ovary syndrome (PCOS), anti-Mullerian hormone (AMH) and sexual dysfunction in obesity and pregnancy in obesity. Immediate positive effects of bariatric surgery are evident at the moment, while for long-term outcomes more prolonged follow-up investigations should be done.

## Introduction

The clinical impact of obesity on female fertility may be expressed through an effect on women’s spontaneous and assisted conception rates, increased miscarriage rates, premature labor, stillbirth and perinatal risks (gestational diabetes and hypertension). Risk of menstrual irregularity and endometrial pathology is increased in obesity [1]. Obesity also increases the need for operative delivery as well as incidence for wound infection and thromboembolic complications [2]. It has been described that excessive body fat may impact the hypothalamic-pituitary-gonadal axis by peripheral and central mechanisms [3]. These perturbations of the hypothalamic-pituitary-ovarian axis may lead to menstrual dysfunction connected with anovulation and infertility [4]. Irregular and prolonged menstrual cycles were more often detected in women with obesity, while luteotropic hormone (LH), follicle-stimulating hormone (FSH), estrogen metabolites and progesterone in urine were lowered [5]. Women with BMI over 25 kg/m^2^ need more time to conceive and it has been demonstrated that overweight and obese women have higher frequency rate of miscarriage, increased need for gonadotrophins and a decreased pregnancy rate [4]. Disorders such as fetal macrosomia, hypertension and gestational diabetes may appear during pregnancy in obesity [6].

Obesity may be managed by conservative (weight loss due to dietary intervention, physical activity, use of weight-reducing drugs) or interventional methods (bariatric procedures). The objective of our investigation was to summarize a reproductive outcome after bariatric surgery in women.

## Material and methods

In this article the reproductive outcomes after the use of interventional methods (bariatric or metabolic surgery) for management of obesity in women are analyzed. Search for original published articles and reviews related to the use of bariatric or metabolic surgery in women has been performed in PubMed using the following search terms (or a combination of terms): obesity, bariatric surgery, female fertility, polycystic ovary syndrome, female sexual function, obesity and pregnancy outcomes. The search was limited to English language articles.

## Bariatric surgery and obesity

Bariatric surgery is indicated for patients aged 18–60 years with a body mass index (BMI) ≥ 40 kg/m^2^ or with a BMI between 35.0 and 39.9 kg/m^2^ and comorbidities, in whom surgically induced weight loss is expected to improve the disorder, but only if lifestyle changes and pharmacological treatment management have been unsuccessful [7–9]. Bariatric procedures are usually classified as predominantly restrictive (gastric binding and sleeve gastrectomy), predominantly malabsorptive (biliopancreatic diversion with or without duodenal switch) and the combination of those two (Roux‑Y gastric bypass) [10]. The bariatric surgery uses results in greater long-term weight loss than the best available nonsurgical interventions for obesity, regardless of the bariatric procedure used [11]. Beneficial effects of bariatric surgery are mainly associated with weight reduction and body weight changes (weight loss or weight regain after bariatric surgery) that are closely associated with the desired outcome of bariatric or metabolic surgery on weight-related comorbidities [12, 13]. Bariatric surgery beneficial effects are associated with reduced food intake, body weight and blood glucose levels, as well as rearrangement of intestinal anatomy leading to markedly elevated concentrations of glucagon like peptide 1 (GLP‑1) and Peptide YY. It is expected that in future, the pathophysiology of bariatric and metabolic surgery approaches and their long-term consequences should be clarified [14]. In future, modulating the release of endogenous GLP‑1 and certain gastrointestinal hormones may be expected to be a promising strategy to mimic bariatric surgery [15].

It is still unknown which procedure is optimal for the weight reduction in obesity, concerning infertility outcome [3]. It is believed that weight loss has a beneficial influence through regulation of menstrual cycles, increase of ovulatory capacity and improvement of fertility in anovulatory overweight or obese women [4].

### Bariatric surgery and hypothalamic-pituitary-ovarian axis

Bariatric surgery may improve the secretion of sex hormones. In a study of 38 morbidly obese women of reproductive age, a significant decrease in estradiol, total and free testosterone and an increase in FSH and SHBG were found 12 months after vertical banded gastroplasty ([16]; Table 1). An increase in LH, FSH and sexual hormone binding globuline (SHBG) as well as a decrease in testosterone and dehydroepiandrosterone sulphate were found after biliopancreatic diversion with duodenal switch [17]. An increase in the urine levels of pregnanediol glucuronide and an increase in serum LH levels, together with a decrease in conjugated estrone, were found after bariatric surgery performed in obese women without PCOS and a BMI around 47 kg/m^2^ [18]. The authors explained postoperative decrease of estrone as a result of diminishing of estrone-producing tissue in adipose tissue after bariatric intervention. A significant reduction in testosterone levels and increased SHBG were found in 100 women after gastric bypass, while other hormones (estradiol, progesterone, LH and FSH) did not differ in comparison with preoperative values [19]. A negative correlation among gonadotrophins and BMI and waist circumference was established among obese female patients [20].

**Table 1:** Reproductive outcomes after bariatric surgery in women

Topic	Outcomes
Bariatric surgery and hypothalamic-pituitary-ovarian axis	Improvement in sex-hormone profile [16]
Bariatric surgery and PCOS	Weight loss; improvement in insulin resistance; decrease of hirsutism score; restoration of menstrual cycles and/or ovulation [27]
Bariatric surgery and anti-Mullerian hormone	Normalization of the AMH levels among obese PCOS women [32]
Sexual dysfunction in obese women and effects of bariatric surgery	Female sexual function resolved after surgical treatment [38]. Weight reduction after bariatric surgery results in reduced sexual dysfunction in female subjects [41]
Bariatric surgery and pregnancy	Bariatric surgery reduces gestational diabetes, pregnancy induced hypertension and macrosomia and increases risk for SGA and prematurity [47, 48]

*PCOS* polycystic ovary syndrome, *AMH* anti-Mullerian Hormone, *SGA* Small for Gestational Age

### Bariatric surgery and PCOS

Polycystic ovary syndrome (PCOS) is a heterogeneous disorder which manifests clinically with symptoms and signs of ovarian dysfunction such as oligoovulation or anovulation, altered ovarian morphology with polycystic ovarian structure and with androgen excess (hirsutism and/or hyperandrogenenemia). In the differential diagnosis it is necessary to exclude hyperprolactinemia and non-classic adrenal hyperplasia. PCOS is the most common disease of the endocrine and reproductive system in women, which is associated with visceral obesity, insulin resistance, impaired glucose and lipid metabolism and elevated cardiovascular risk factors [21]. The diagnosis of PCOS is made on the basis of the existence of three specific criteria: oligo-anovulation, clinical or biochemical signs of androgens excess, and ultrasound picture assessment of ovarian polycystic morphology [22]. According to the Rotterdam criteria, two of three diagnostic criteria are necessary for the diagnosis of PCOS [23]. Insufficient response to infertility treatment and increased pregnancy complications risk are common findings among obese PCOS women [24]. A rise of obesity in PCOS patients may induce the appearance of reproductive and cardiovascular disease [25]. Bariatric (metabolic) surgery remains as a final approach in PCOS cases where previous lifestyle and pharmacological management did not produce satisfactory results [26]. Earlier, it was found that bariatric surgery use in obese PCOS women may induce a reduction in body weight, a decrease in insulin resistance, a correction of the hirsutism score and improvements in menstruation and ovulation ([27]; Table 1). Ovulation was established in 71% in a study of 195 anovulatory obese patients after surgery intervention. Greater weight loss was associated with ovulation regain [28]. A meta-analysis of 29 studies published up to June 2016 was done in order to establish the presence of the disorders of gonadal function related to obesity and to assess the therapeutic response after bariatric surgery on this disorders and sex hormones [29]. It was found that obesity-related disorders of gonadal function were very prevalent, with 36% expressed as PCOS. Resolution of PCOS was detected among 96% of them. Increase of SHBG and a decrease of estradiol and testosterone together with an improvement in hirsutism and menstruation appeared as a result of bariatric surgery. The authors concluded that bariatric surgery should be used as a first choice for the management of morbidly obese patients with disorders of gonadal function [29]. In a large retrospective study that included 930 subjects after bariatric interventions, 44 patients with PCOS were compared with 65 controls. Significant weight loss with a significant decline in total testosterone and free testosterone appeared after bariatric surgery in PCOS patients, with simultaneous reduction of hyperandrogenism and menstrual irregularity. As a result of bariatric surgery, significant weight loss with a decrease in total and free testosterone were achieved [25]. There is still an unsolved question among experts as to which method of bariatric surgery is best to apply in severely obese PCOS patients [26]. The same authors suggest that AMH changes after the intervention in PCOS patient may be associated with the increase in conception rate.

### Bariatric surgery and anti-Mullerian hormone

The measurement of the AMH or Mullerian inhibiting substance (MIS) could be useful for the assessment of the ovarian reserve [30]. Considerably lower levels of AMH and inhibin B were detected in 36 obese women without PCOS, aged 42–45 years in comparison to normal weight late reproductive age women. There was no difference between antral follicles number and volume of ovaries [31]. The authors suggested that the lower anti-Mullerian hormone may be the consequence of physiologic events rather than the decrease of ovarian reserve. Chiofalo et al. demonstrated elevated AMH levels in PCOS patients, no matter what their body weight was. Correction of AMH may be used as predictor for better fertility only in obese PCOS. The authors suggested that changes in AMH levels may be associated with decrease in body weight ([32]; Table 1). AMH levels were studied in 16 women before and after bariatric surgery and its significant decrease was detected in patients under 35 years of age. This decrease was not found in women older than 35 years. As explanation it was supposed that AMH genes expression could be affected during surgery or it may be a consequence of surgical stress [33]. Beneficial effects of bariatric surgery are demonstrated on normalization of menstruation and AMH levels in severely obese PCOS [34]. Repeated measurements of AMH levels after bariatric surgery were performed in a few studies. The impact of sleeve or gastric by-pass interventions on ovarian reserve was explored in 39 patients. AMH levels were measured before and 6 and 12 months after surgery. A strong decrease in AMH values was detected 12 months after surgery without a relation with loss in weight and the authors of the study postulated a negative influence of surgery on the ovarian reserve [35]. They hypothesized that postoperative stress of growth follicles in ovaries may be the reason for the reduction in AMH values, indicating the need for prolonged observational studies in order to clarify observed changes. In another study, the analysis of AMH values was performed in 53 patients with obesity before and 3 and 6 months after sleeve gastrectomy. There was no difference in AMH between basal and 3‑month values, while a significant increase in AMH values was found after 6 months [36]. These studies have evaluated only the short-term evolution of ovarian function and fertility improvements after bariatric surgery, which are not reported in some of them, so that prolonged observational studies are necessary concerning influence of bariatric surgery on the function of the ovaries. Recently, a national prospective multicentre cohort study was started in December 2020 in which AMH levels would be measured before surgery, 12, 24 and 36 months after bariatric surgery in the bariatric surgery obese group and at baseline and 12, 24 and 36 months in the control group. Authors postulated that AMH level is known to decrease with age and the speed of that evolution is mostly unknown in the obese group and they expect that the deterioration of the ovarian reserve after bariatric surgery will be defined according to the natural evolution of the AMH level observed in the control group. Study completion date is expected to be in March 2025 [37].

### Bariatric surgery and sexual dysfunction in obese women

Obesity increases the probability of sexual disorders. Literature data indicate that the application of a female sexual function index (FSFI) questionnaire and its analysis may show that female sexual dysfunction (FSD) resolved after surgical treatment [38]. In contrast, a study was published where FSD did not differ preoperatively and postoperatively, while desire and arousal were increased significantly postoperatively [39]. It was also reported that the participants’ FSFI scores did not differ in comparison with controls after surgery [40]. In a study where sexual satisfaction levels among 60 obese women prior to and following bariatric surgery were validated using the FSFI, baseline sexual function was significantly lower among obese preoperatively in comparison to non-obese women. Before the procedure, 51.6% exceeded the cut-off for FSD, at 6 months 39.5% exceeded the cut-off and at 12 months postoperatively 41.9% exceeded the cut-off. There was no significant difference in sexual satisfaction levels between 6‑month and 12-month values of FSFI in follow-up among obese women and the authors concluded that weight reduction after bariatric surgery results in reduced sexual dysfunction in female subjects ([41]; Table 1).

### Bariatric surgery and pregnancy

Presence of obesity before pregnancy poses a significant risk of adverse maternal and perinatal outcomes [42]. Excess body mass in the course of pregnancy may lead to development of complications in mother and child [43]. Obesity in combination with pregnancy may result in birth defects, pre-eclampsia, gestational diabetes, stillbirth and cesarean deliveries [44]. Significant weight loss and fertility improvement was observed after the application of bariatric surgery among an obese female population [44]. A significantly higher rate of cesarean section was reported among postbariatric surgery patients [45]. Reproductive function after gastric bypass surgery is characterized by a shortened follicular phase and improved female sexual function [46]. Reduction of gestational diabetes, pregnancy-induced hypertension and macrosomia and increase in SGA and prematurity was observed after bariatric intervention ([47, 48]; Table 1). Presence of excess body mass of the mother and gestational diabetes may indicate an impaired metabolic adaptation. Disorders of gene regulation of placental size and function and endocrine sensitivity were observed in cases of excess maternal body mass and gestational diabetes [49]. Gestational diabetes mellitus increases the risk of large-for-gestational age fetus, due to increased fetal insulin secretion associated growth properties [50]. Early studies demonstrated reduced incidences of gestational diabetes after delivery among women with previous bariatric interventions in comparison with those who had delivery before bariatric surgery [51]. Reduced risk of gestational diabetes and LGA and increased risk for SGA as well as shorter gestation were found [52]. In the same study, no difference was found in the frequency of congenital malformations among groups [47]. Recently, a significant decrease of gestational diabetes mellitus after bariatric surgery was also found in a French matched-cohort study [53]. Avoidance of pregnancies are suggested during the rapid weight loss after bariatric intervention, together with the use of adequate contraception [54], and 12–18 months after bariatric intervention a pregnancy is not recommended in order to enable adequate loss of body weight and its maintenance together with corrections of nutrient content and electrolyte disorders [43, 55]. Weight loss may be rapid (after gastric bypass and sleeve) and slower (after gastric banding). Two common surgical complications during pregnancy after bariatric surgery may be found: gastric herniation and gastric band slippage [43].

The risk for gestational diabetes was found to be lower after gastric binding in comparison to gastric bypass. The risk for surgical complications was lower for pregnancies after gastric banding in comparison with gastric bypass and sleeve. Birthweight was higher for new-borns after gastric banding in comparison with gastric bypass [56]. In a large study performed in the USA, the LABS-2 study, weight loss after a bariatric intervention was not decreased with a subsequent pregnancy, while prepregnancy surgical intervention (laparoscopic adjustable gastric banding and Roux-en‑Y gastric bypass) was not associated with differences in offspring birth weight [57]. In another study, women who were pregnant after bariatric intervention were compared to controls, with the aim to investigate the effect of restrictive and malabsorptive approaches on the outcome of pregnancy, pregnancy-induced diseases and perinatal outcomes. Patients after bariatric procedures had significantly less gestational diabetes and preterm delivery and less pregnancy-induced hypertension but the rate of cesarean section was increased; the risk for SGA was increased and the risk for LGA was decreased [58]. Recently, a Swedish nationwide examination was performed to determine major birth defects in infants of mothers who underwent Roux-en‑Y gastric bypass surgery prior to pregnancy vs. infants of women without previous bariatric surgery [59]. Less major birth defects were detected among newborns of mothers with gastric bypass surgery in comparison with controls. The major heart defects were responsible for 60% of birth defects in the same population. No cases of neural tube defects were detected in the same group. As a potential explanation for these findings, the authors suggested better metabolism of glucose and some physiological improvements occurring after surgery. Study of pregnancy outcomes after twin gestations, documented more positive pregnancy outcomes among women who had undergone bariatric surgery procedures with less gestational diabetes and gestational hypertension [60]. Pregnancy following gastric banding negatively affects postoperative final weight loss [61]. Negative effect of both sleeve and gastric by-pass on fetal growth has been reported [62]. A deficiency or insufficiency of 25 (OH) D vitamin was found in the course of pregnancy in a woman with previously performed gastric by-pass [63].

Del Sordo et al. performed a study of postnatal health and short-term and long-term outcomes of children from pregnancies after various bariatric interventions [64]. They identified 5.4% underweight, 59.5% normal weight, 16.2% overweight and 18.9% obese infants. The prevalence of neurodevelopmental disorders was higher if the pregnancy occurred in less than 18 months after bariatric surgery and if they were small for gestational age at birth. Children born after biliopancreatic diversion were more often overweight, while children born after Roux-en‑Y gastric bypass showed a higher rate of normal weight. Atopic dermatitis was more often developed in newborns of mothers who previously underwent biliopancreatic diversion [64].

## Conclusion

Bariatric procedures in women, at the moment, represent a satisfactory treatment of obesity and associated comorbidities, when previously used methods (diet, physical exercise, and pharmacological intervention) did not produce the expected results. Data from literature indicate that bariatric surgery may improve the profile of sex hormone secretion. Significant weight loss and a fertility improvement was observed after application of bariatric surgery among obese female populations. Loss of weight, decrease in insulin resistance, improvement of hirsutism score, normalization of AMH values and establishment of menstruation and ovulation were found after use of different bariatric surgery methods in obese women with PCOS; however, the question of the most suitable surgical approach for obese PCOS patients has no answer yet. Women with previous bariatric surgery have a risk for some adverse perinatal outcome, requiring specific nutrition recommendations before and during the pregnancy, together with adequate follow up of the fetal growth and development. Reduced sexual dysfunction was detected after surgical weight loss. Currently, immediate bariatric surgery results are positive, while for long-term outcomes we need more studies, more patients and their prolonged follow-up.
